# Understanding Trap Effects on Electrical Treeing Phenomena in EPDM/POSS Composites

**DOI:** 10.1038/s41598-018-26773-y

**Published:** 2018-05-31

**Authors:** Boxue Du, Jingang Su, Meng Tian, Tao Han, Jin Li

**Affiliations:** Key Laboratory of Smart Grid of Education Ministry, School of Electrical and Information Engineering, Tianjin University, Tianjin, 300072 China

## Abstract

POSS (polyhedral oligomeric silsesquioxane) provides an interesting alternative nano-silica and has the potential of superior dielectric properties to restrain electrical degradation. By incorporating POSS into EPDM to suppress electrical tree, one of precursors to dielectric failure, is promising to improve the lifetime of insulation materials. This paper focuses on the electrical treeing phenomena in EPDM/OVPOSS (ethylene propylene diene monomer/octavinyl-POSS) composites based on their physicochemical properties and trap distributions. ATR-IR and SEM characteristics are investigated to observe the chemical structure and physical dispersion of EPDM/OVPOSS composites. Electrical treeing characteristics are studied by the needle-plane electrode, and the trap level distributions are characterized by surface potential decay (SPD) tests. The results show that the 3 wt% EPDM/OVPOSS is more effective to restrain the electrical tree growth than the neat EPDM in this paper. It is indicated that the EPDM/OVPOSS with a filler content of 3 wt% introduces the largest energy level and trap density of deep trapped charges, which suppress the transportation of charge carriers injected from the needle tip and further prevent the degradation of polymer molecules. The polarity effects are obvious during the electrical treeing process, which is dependent on the trap level differences between positive and negative voltage.

## Introduction

Electrical tree degradation is a key issue to cause failures of cable accessories at both distribution and transmission voltage levels^1^. EPDM is the main insulation material in joints and terminals of cable accessories^2,3^. As the two-phase interface or electric field concentration exists in the cable accessories, EPDM becomes susceptible to degrade in HVDC or HVAC cable transmission system^4^. The joint failure was occupied for 21% of the cable system^5^. The electrical tree inception voltage with AC voltage was lower than the pulse voltage one, which was induced by the alternate injection of carriers to release more energy^6^. No trees appeared with DC voltage of 70 kV or −60 kV, while the electrical tree was observed at 35 kV with pulse voltage. This phenomenon was caused by the homo space charges, which were accumulated around the needle electrode to modify the electric field distribution with DC voltage^7^. The pulse voltage with higher frequency accelerated electrical tree to propagate, the tree length tended to be longer and the deterioration area became larger^8^. Different methods were employed to enhance the electrical tree resistance in high voltage cables, such as adding treeing inhibitors and modifying the insulation material^9,10^. Results shown that nanofillers were superior in inhibition of electrical treeing in polymeric insulating material^11–13^. A small quantity of alumina nano-fillers improved the tree inception voltage and restrained tree growth of the LDPE polymer material^14^. The presence of nanoparticles inside the tree channel was shown to be the reason for the increased treeing resistance of epoxy nano-composites^15^. Tanaka *et al*. gave a deep insight into the microscopic interfaces between nanofillers and polymer matrix, electrical treeing resistance mechanism was revealed by the multi-core model of the polymer-nanofiller interaction zones^16^.

The electrical performance of nano-composites was dependent on the uniform distribution of nanoparticles, which affected the trap distribution in the bulk and lead to the change in carrier mobility characteristics^17^. Some results verified that the deep traps were introduced in nanocomposites such as XLPE/SiO_2_, LDPE/ZnO and PI/TiO_2_ nanocomposites^18–20^, and it suggested the property of deep traps had a strong relation with the dielectric failure. Electrical tree was a mid-term or long-term breakdown phenomenon and it was associated with the trap distribution of polymers. During the electron collision and recombination process, hot electrons transported in the polymer matrix and collided with the molecular chain to generate new tree channels. Deep traps could capture mobile charges or induce them hardly to detrap. In addition, the homocharge accumulation caused by the deep traps near the electrodes could suppress charge injection. Thus, the mobility and density of charge carriers were reduced^21^, which weaken the electrical tree destruction for the insulation materials. It was reported electrical tree inception was affected by the free volume, where the injected electron was accelerated to collide with free volume wall, producing free radical and breaking polymer bond^22^. However, the injected electrons were quickly trapped due to the large localized states introduced by nanofillers, the electrons were not easy to reach the energy threshold in the free volume^23^. Consequently, the electrical breakdown performance was improved in nano-composites.

POSS has recently attracted considerable attention for its special structure. In POSS, the bond length of Si—Si is 0.5 nm, which features an interesting alternative nano-silica cage-structure in nanometer scale size^24^. The inorganic cage-structure is rigidity and stability. The organic side-groups can self-assemble to act as “anchor point”, or react with the polymer matrix to act as a coupling agent, resulting in a reconstruction of the polymer network on a nanometric scale^25^ to improve the mechanical stability of the matrix. Adding POSS to polymers brought about significant improvements in the properties such as increased thermal stability, oxidative resistance and dielectric properties^26,27^. The introduction of small fraction of POSS (i.e., 2.5%) resulted in a little homo charges accumulation near the ground electrode^28^. OVPOSS has eight vinyl double bonds at every corner of the cage-structure, which can react with the EPDM molecular chain to form a crosslink structure with the help of 2,5-dimethyl-2,5-di-(tert-butylperoxy)-hexane^29^. Thus, OVPOSS is superior to octamethyl POSS, octaisobutyl POSS and trisilanolphenyl POSS, which are not easy to chemically bonded to the polymer matrix. Compared with the reaction between the vinyl double bonds of OVPOSS and the polymer matrix to form regular particles, SiO_2_ is easy aggregated together without the surface modification. OVPOSS shows the advantages of forming organic–inorganic hybrid nanocomposites with a variety of structures, such as linear, star-shaped as well as network types^30^. OVPOSS can play a role of coagent and filler-reinforcing agent for EPDM because of the high Si-O bond energy and its cage-structure nanometer effect. However, few studies on the electrical treeing phenomena and trap distribution of EPDM/OVPOSS have been carried out until now, and the electrical tree resistance mechanism is still unknown considering the traps distribution introduced by OVPOSS.

In this research, EPDM is employed as polymer matrix, which is widely used as insulating material in cable accessories. EPDM/OVPOSS with the filler content of 0 wt%, 1 wt%, 3 wt%, 5 wt% and 10 wt% are prepared by melt blending. To observe the chemical structure and physical dispersion of EPDM/OVPOSS composites, ATR-IR (attenuated total reflectance infrared spectroscopy) and SEM (scanning electron microscopy) experiments are employed. The electrical treeing properties of EPDM/OVPOSS, including the tree morphologies, inception probability and growth characteristics are researched. Meanwhile, their trap level distributions are characterized by surface potential decay (SPD) tests. Based on the results, a schematic model is proposed for illustrating the mechanism of traps restraining electrical treeing, thereby further revealing the relationship between the trap level distributions and the electrical properties.

## Results and Discussion

### ATR-IR and SEM characteristics of EPDM/OVPOSS samples

**Figure 1:**
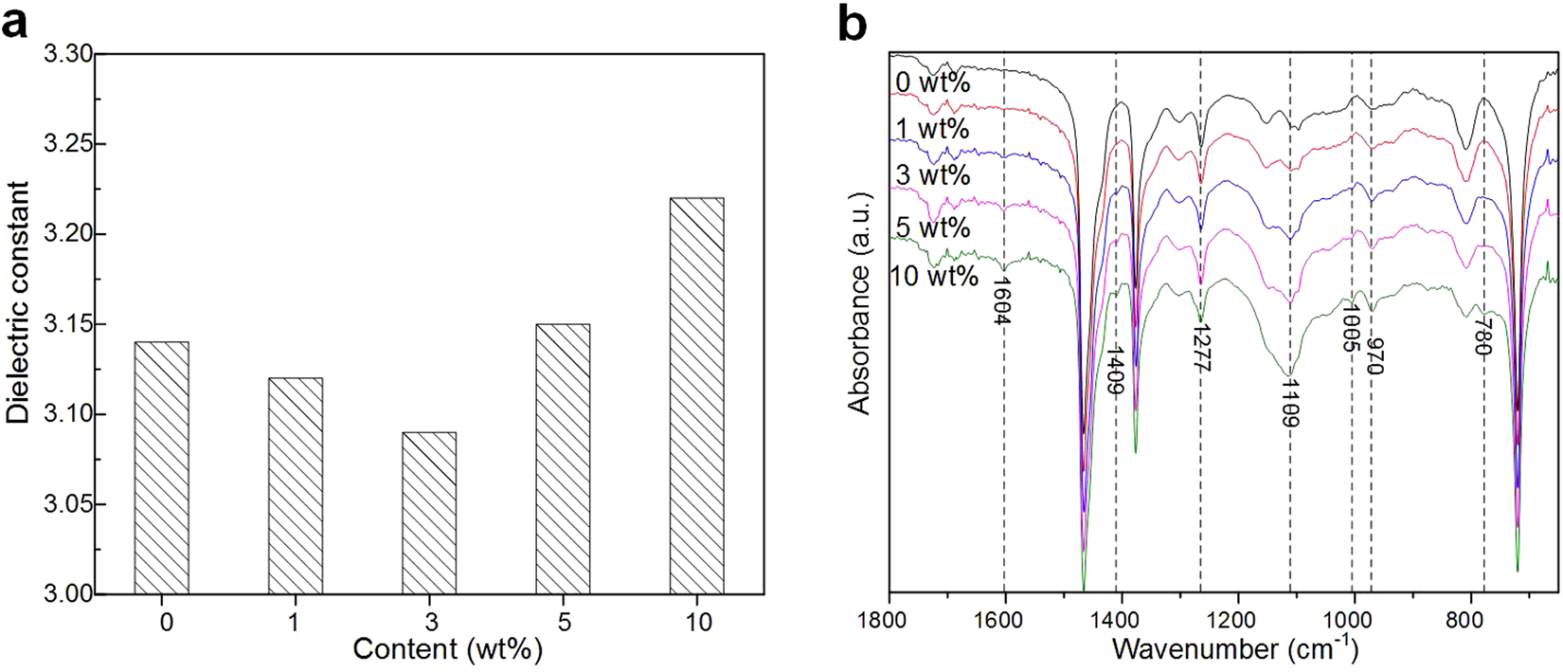
(**a**) Dielectric constant as a function of OVPOSS content. (**b**) ATR-IR of EPDM/OVPOSS composites.

Fig. 1a presents the dielectric constant as a function of OVPOSS content at 25 °C. The dielectric constant of the neat EPDM is ~3.14, it is larger than the EPDM/OVPOSS with a filler content of 1 wt% and 3 wt%, which is respectively ~3.12 and ~3.09. As the OVPOSS content reaches 5 wt% and 10 wt%, the dielectric constant is respectively ~3.15 and ~3.22, which is even larger than the neat EPDM one. The slight dielectric constant decrease of EPDM/OVPOSS with a filler content of 1 wt% and 3 wt% is attributed to the intrinsic low dielectric constant of POSS, which is about 2.1~2.7 for the lower density and larger pore size of the cage-structure in OVPOSS. Moreover, the OVPOSS is with a symmetric molecular structure to mainly exhibit electronic polarization, the crosslink between the vinyl double bonds of OVPOSS and EPDM will restrict the movement of polar groups. The increase dielectric constant of EPDM/OVPOSS with a filler content of 5 wt% and 10 wt% is resulted from the remaining polar groups during the cross-linking process, or the exposure of the polar Si-O-Si core due to the insufficient shielding from its short -CH=CH_2_ by the redundant OVPOSS as discussed in the following sections.

ATR-IR is carried out to demonstrate the chemical structure of EPDM/OVPOSS samples in Fig. 1b. The ATR-IR peaks at 970 cm^−1^ and 1005 cm^−1^ represent the -CH_2_ and –CH out of plane, while the ATR-IR peaks at 1277 cm^−1^ and 1409 cm^−1^ represent the -CH and –CH_2_ in plane^31^. The ATR-IR peaks at 1109 cm^−1^ and 780 cm^−1^ represent the vibrations of Si-O-Si and Si-C, which are slightly increased with increasing OVPOSS content. The ATR-IR peak at 1604 cm^−1^ represents the vinyl group, which is less intensity when the OVPOSS content is lower than 3 wt%. The result suggests that most vinyl groups of OVPOSS are self-assembled or reacted with EPDM to endow a strong interaction during the crosslink reaction process. As the OVPOSS content is larger than 5 wt%, the peaks at 1604 cm^−1^ is slightly increased, which indicates the OVPOSS raw material can be existed in the EPDM/OVPOSS composites.

**Figure 2:**
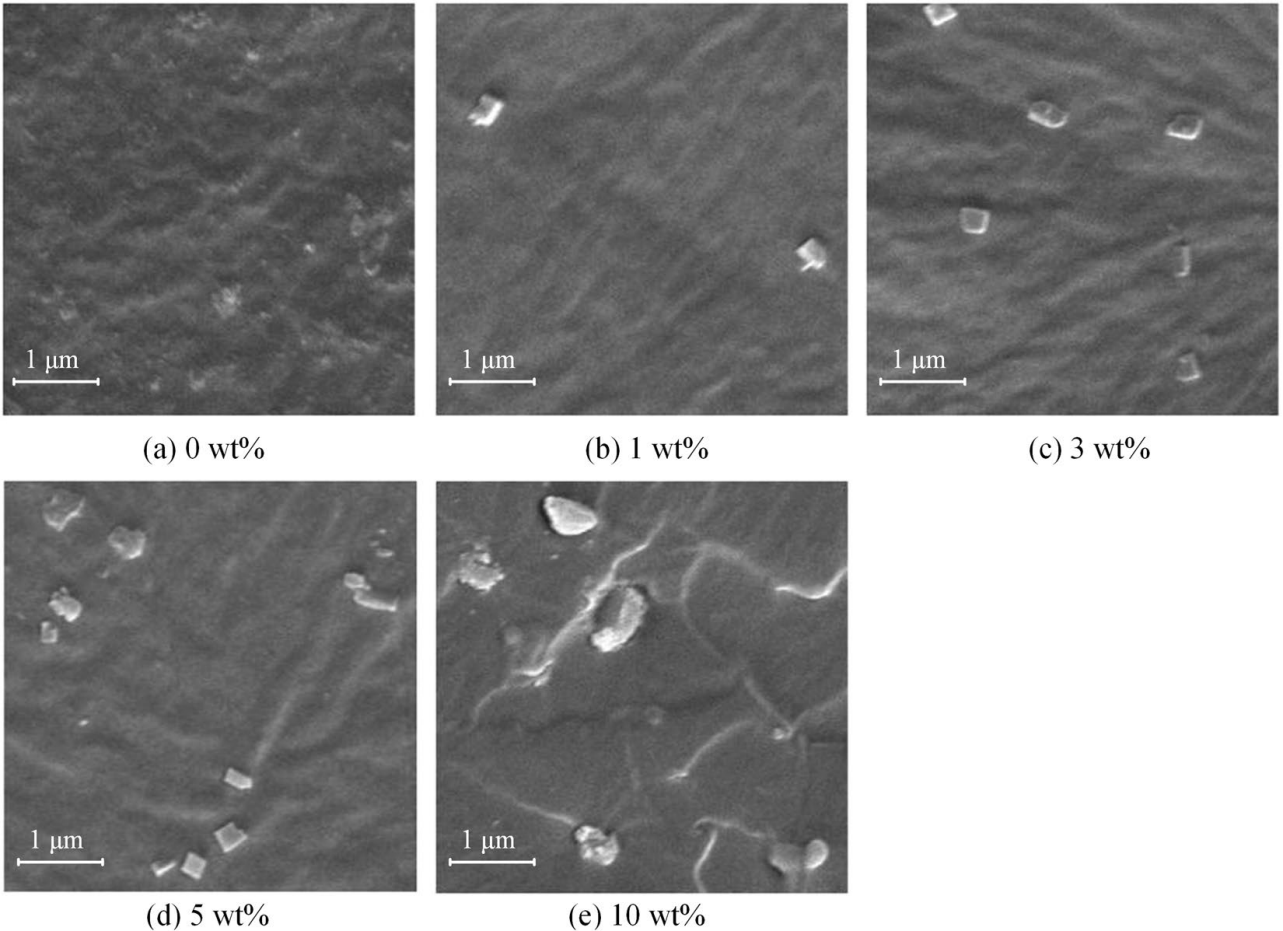
SEM images of EPDM/OVPOSS composites.

To observe the dispersion of OVPOSS, the typical images are characterized by SEM analysis as shown in Fig. 2. In Fig. 2b,c, OVPOSS is self-assembled in quasi-rectangular shape and well dispersed in nanometer scale, OVPOSS is much more sparsely in Fig. 2b. Even though the OVPOSS is not dispersed in molecular level, the regular particles can act as “anchor point” to improve the mechanical stability for the chemical bonds between the polymer matrix and OVPOSS. Moreover, the particles can enhance the electrical stability of EPDM for its nanometer effect and the special cage inorganic core. In Fig. 2d, OVPOSS is dispersed in irregular patterns: strip shapes, quasi-rectangular or sphere shapes. OVPOSS is aggregated together, which leaves a large space in the center region. In Fig. 2e, OVPOSS is severely aggregated, the shape is irregular and the size is ~1 μm. By combination the results of ATR-IR and SEM, the EPDM/OVPOSS physical features are clarified with different OVPOSS content.

### Electrical tree degradation

**Figure 3:**
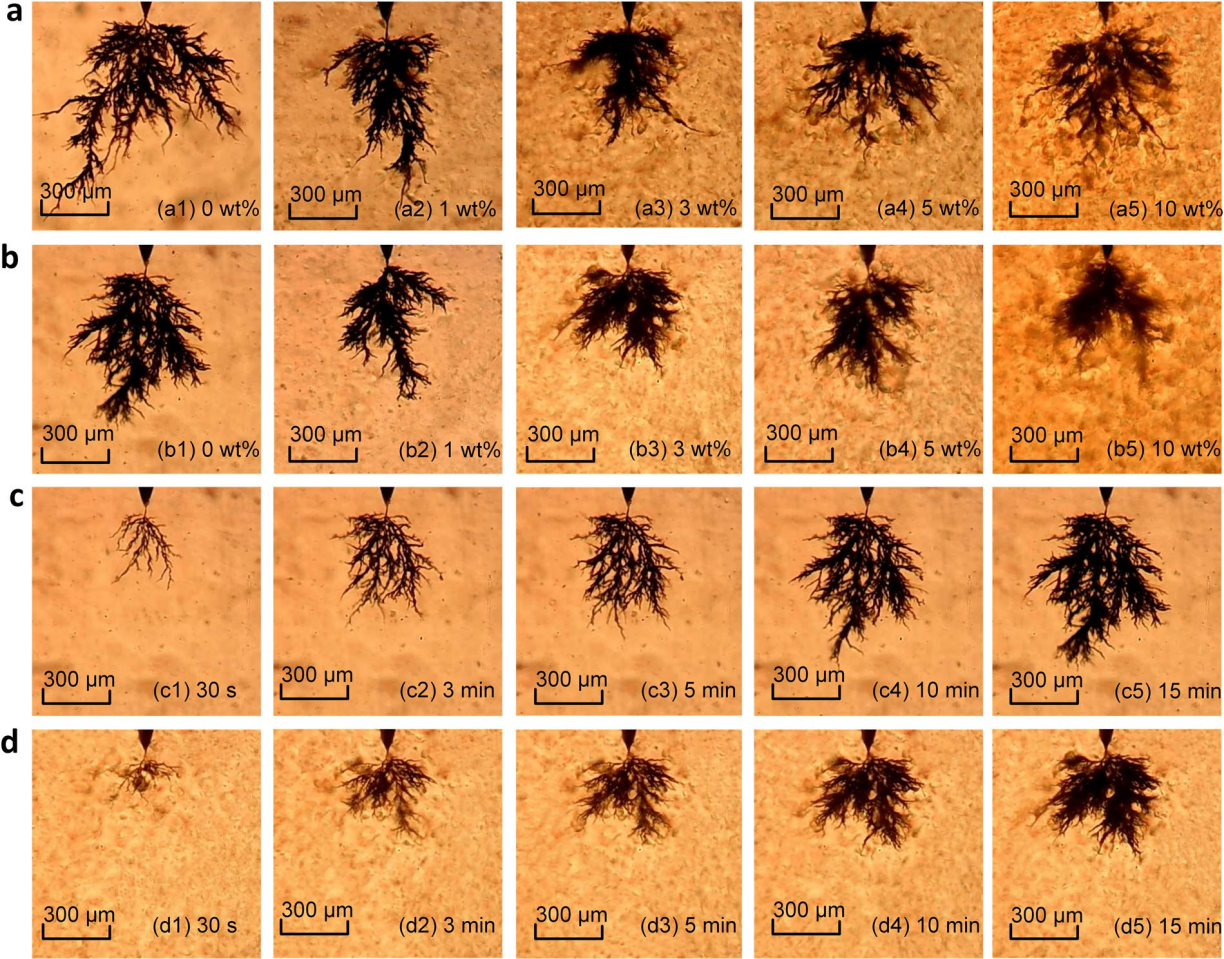
(**a**) and (**b**) are the electrical tree morphologies after 15 minutes, corresponding to +25 kV and −25 kV respectively. (**c**) and (**d**) are the electrical tree morphologies at different time with −25 kV, corresponding to the neat EPDM and 3 wt% EPDM/POSS respectively.

With widespread application of high-capacity, high-voltage and high-frequency power electronic equipment in power systems, the electrical properties of insulation materials are faced new challenges in HVDC cable system. Lightning intrusion, switching surge or commutation failure can be generated in HVDC cable system, which induce repetitive pulse voltage to directly trigger failures or indirectly cause failures by accumulative effect for insulation materials. To investigate the accumulative effect of pulse voltage on EPDM/OVPOSS, the pulse voltage with frequency of 200 Hz was employed in this paper. The different appearance of electrical tree morphologies is related to many factors, such as nanofillers and electric field^14^. As the electrical tree is rapidly initiated after the pulse voltage of 25 kV is stressed on the samples, the electrical tree structures are investigated in the same time. Fig. 3a,b show electrical tree morphologies with different filler content of OVPOSS after 15 minutes, corresponding to +25 kV and −25 kV respectively. From a1 to a5, the electrical tree with different morphologies are observed for the neat EPDM and the filled EPDM composites. In the neat EPDM, the tree morphologies is bush-branch type, which includes the bush-type pattern around the needle tip and some branch-type pattern propagating to the plane electrode. The extended longer branch-type channels reduce the durability and reliability of cable accessories and shorten the lifetime of the cable system. When the OVPOSS content is 3 wt%, there is only bush-type pattern with the shortest tree length. Whereas the filler content exceeds 3 wt%, a more sparsely electrical tree pattern with the longer tree channels are observed, even though the tree morphologies is kept bush-type. The electrical tree growth trend in Fig. 3b is almost the same with that in Fig. 3a except a shorter tree length is observed in Fig. 3b. The different electrical tree propagation characteristics are attributed to different trap distribution and carrier mobility behaviors under positive and negative voltages as shown in the following sections. To in-depth study the electrical tree growth characteristics at different stages, the tree morphologies are observed in the neat EPDM and the filled EPDM composites. Fig. 3c,d show tree morphologies at different time with −25 kV, which are the neat EPDM and 3 wt% EPDM/POSS respectively. The electrical tree is shorter with less tree channels in unfilled samples than the filled one at the initiation stage, which means the OVPOSS can restrain the tree propagation at this stage. Compared with unfilled EPDM in Fig. 3c, the OVPOSS sustains to inhibit electrical treeing after the tree initiation as shown in Fig. 3d.

The electrical tree inception process is associated with the formation of low-density region^32^, where the chain scissions are more easily to occur when charge carriers are accelerated by electric field to gain sufficient kinetic energies. The charge carriers are either injected from the needle tip or excited via an Auger-type process^32^ when non-radiative transition of energy is occurred by trapping and recombination of charge carriers. This process can be expressed as:

1 $$\begin{array}{c}AB+{e}^{-}(hot)\to {A}^{\ast }+{B}^{\ast }+{e}^{-}(cold)\\ {\rm{or}}\to {A}^{\ast }+{B}^{\ast }+{e}^{-}(trapped)+energy\,release\end{array}$$

Charge carriers can be captured by deep traps, thus the charge mobility is decreased^33^. In the filled EPDM, the trap distribution and carrier mobility behaviors are different from the unfilled composites^21^, which can result in different electrical tree inception characteristics.

**Figure 4:**
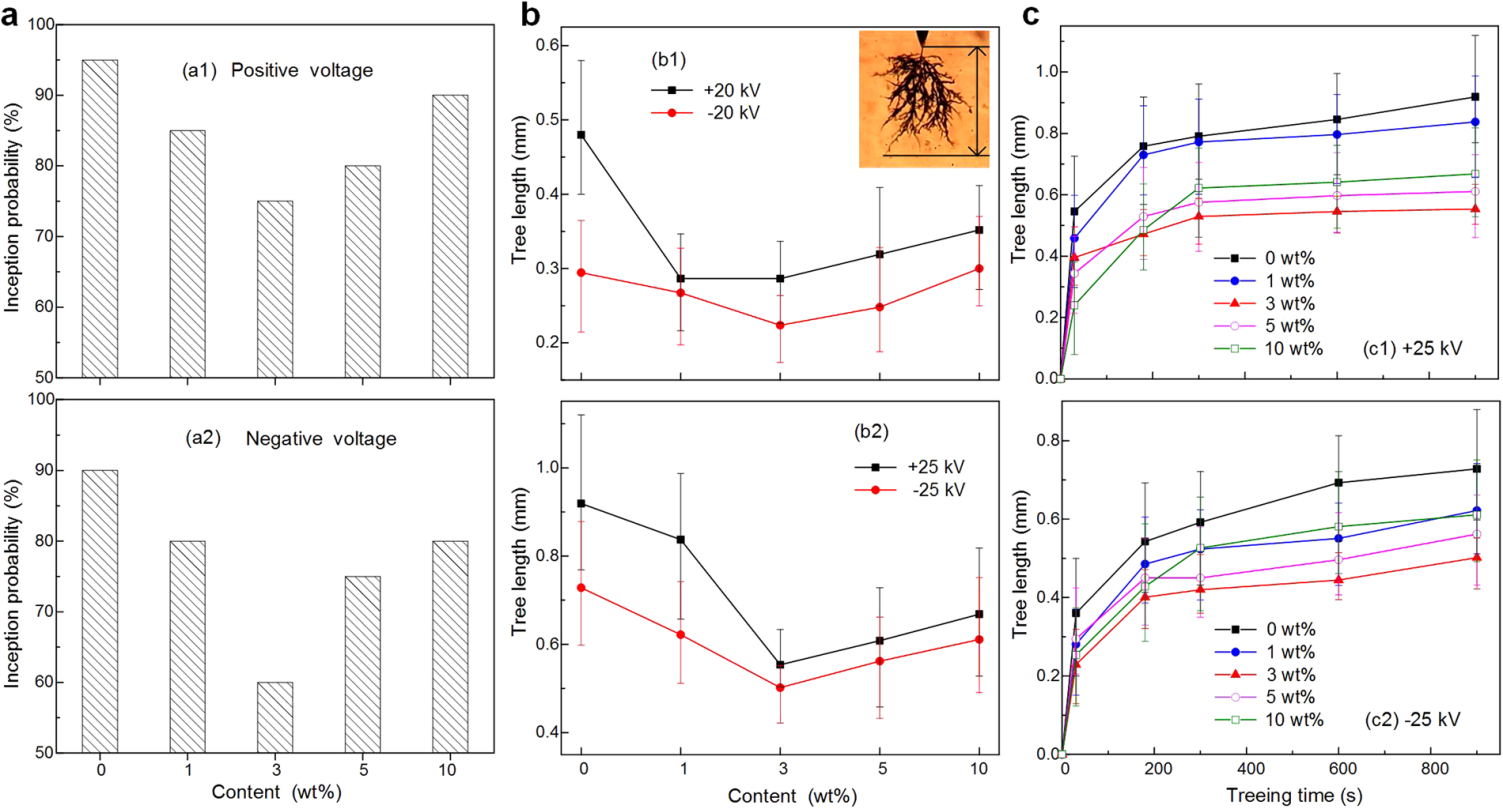
(**a**) Electrical tree inception probability as a function of OVPOSS content, (a1) and (a2) is with +17 kV and −17 kV respectively. (**b**) Electrical tree length as a function of OVPOSS content, (b1) and (b2) is with ±20 kV and ±25 kV respectively. (**c**) Electrical tree length as a function of treeing time at different POSS content, (c1) and (c2) is with +25 kV and −25 kV respectively.

The electrical tree initiation is defined as the tree length of 50 μm is observed. The electrical tree inception probability is referred to the proportion of samples with electrical tree structure, 20 samples are tested in 5 minutes. In the research, there is no observed electrical tree structure as the pulse voltage is less than 15 kV in 60 minutes. Almost all the samples are observed electrical tree structure in less than 1 minute with the pulse voltage of 20 kV. The electrical trees are quickly gone to breakdown when the pulse voltage reaches 30 kV. Therefore, the pulse voltage of 17 kV is chosen to investigate tree inception probability, 20 and 25 kV are selected to clarify the electrical tree propagation tendency. Fig. 4a shows electrical tree inception probability as a function of OVPOSS content, a1 and a2 are with +17 kV and −17 kV respectively. The electrical tree inception probability of EPDM/OVPOSS with the content of 0 wt%, 1 wt%, 3 wt%, 5 wt% and 10 wt% is respectively 95%, 85%, 75%, 80% and 90% when the samples are tested with positive voltage. The tree inception probability tendency tested with negative voltage is similar to the positive voltage one except a smaller value.

The electrical tree propagation period consists of tree extension and trunk widening by erosion due to partial discharge attack in tree channels^34^. The tree length indicates the tree extension characteristics. When the longest electrical tree channel reaches the ground electrode, the insulation failure is occurred. Therefore, tree length can reflect the electrical tree breakdown resistance. Tree length is referred to the distance between the needle tip and the end of the longest tree branch along the electric field direction, which is indicated in the top right in Fig. 4b1. Tested for 15 minutes, electrical tree length as a function of POSS content is shown in Fig. 4b, b1 and b2 are with ±20 kV and ±25 kV respectively. After 15 minutes, the bush tree stays in the stagnation stage for a long period until a quick breakdown occurs. When the filler content increases from 0 wt% to 3 wt%, the tree length becomes shorter, indicating that the fillers restrain the tree extension along the electric field. As the filler content continues increasing, the tree length grows a little longer, which is considered to be caused by the agglomeration of OVPOSS. The aggregation of the nanofillers could weaken the electrical tree resistance, which affected the trap distribution, conduction current and space charge^35^. To fully understanding the relation between the tree length and treeing time, electrical tree length as a function of treeing time at different OVPOSS content is demonstrated in Fig. 4c, c1 and c2 are with +25 kV and −25 kV respectively. In the initiation stage of the unfilled EPDM, the tree channel rapidly reaches ~0.8 mm in a few minutes and then keeps a very slow growth speed with positive voltage. The tree length becomes shorter in the filled EPDM, and the shortest tree length is obtained when the OVPOSS content is 3 wt%. Even though the tree length growth tendency tested under negative voltage is similar to the positive voltage one, the tree length is much shorter under negative voltage. The results shown in Fig. 4 indicates that the reasonable OVPOSS content acts as “degradation inhibitor” and suppresses the electrical treeing in insulating materials to improve the lifetime of polymer dielectrics.

### Trap distribution and carrier mobility behaviors

**Figure 5:**
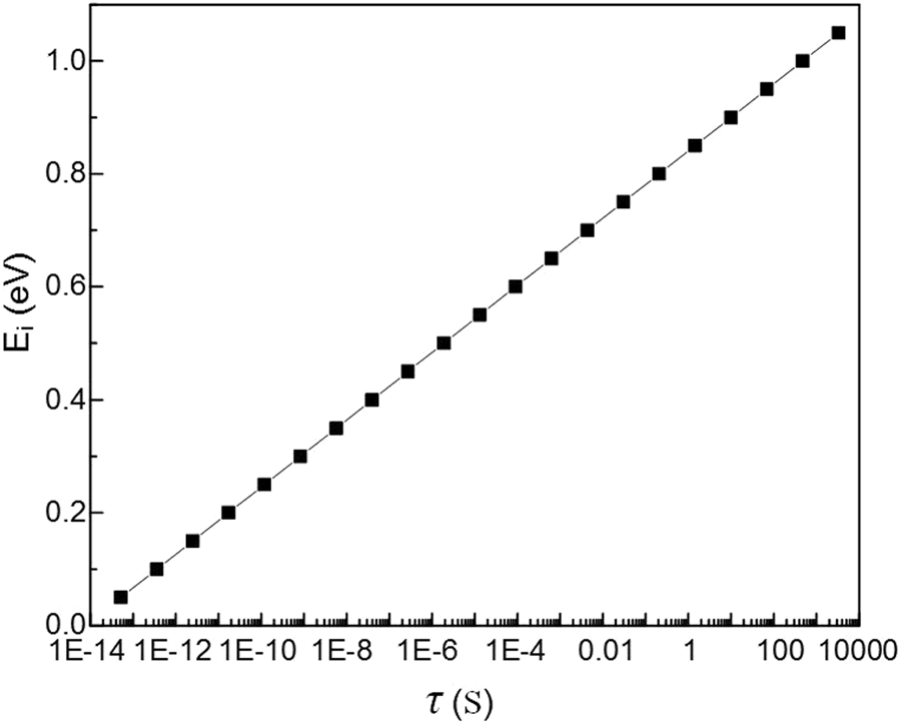
Residence time of charges in different trap energy levels.

The residence time (*τ*/s) of the charge in traps can be estimated by using a two-potential model. In the model, the two-well minima are divided by a barrier of height *E*_*i*_, which is corresponded to the trap energy. The *τ* can be expressed as^36^:

2 $$\tau =\frac{1}{{\Gamma }_{AB}}$$

3 $${\Gamma }_{AB}={v}_{0}\exp \,[\,-\,{E}_{i}e/kT]$$

where *Γ*_*AB*_ represents the hopping probability from well A to B, *k* = 1.38 × 10^−23^ J/K is the Boltzmann’s constant, *T* = 300 K is the temperature, *v*_0_ = 1.336 × 10^14^ s^−1^ is the attempt frequency. Then the relation between the *τ* and *E*_*i*_ is shown in Fig. 5. Though the shallower trap energy levels less than 0.7 eV are not observed by the SPD method considering the switching off speed of voltage source, the shallow trap energy levels ranged from 0.7 eV ~0.9 eV are observed. Moreover, the trap energy levels larger than 0.9 eV are all observed, which is regarded as deep traps to improve the breakdown properties of insulation materials.

The polymeric materials contain chemical defects, which are introduced by cross linking or bond breaking. Meanwhile, there are physical defects in polymeric materials, which are imported by impurities or structural relaxation. The chemical and physical defects can serve as traps to capture charges and reduce the mobility of charges. The traps energy is ranged from 0 to a few eV. In polymeric materials, the chemical defects originated from OH, nonconjugated C=C or C=C=C and the physical defects present the shallower energy level. The chemical defects originated from C=O and conjugated C=C constitute the deeper energy level^36,37^. OVPOSS features an interesting alternative nano-silica core and organic side-groups, which is not only shown a special electrostatic potential distribution, but also reacted with the polymer matrix to modify the configuration of EPDM system. Therefore, the trap distribution behaviors are modified as the OVPOSS is filled into EPDM.

**Figure 6:**
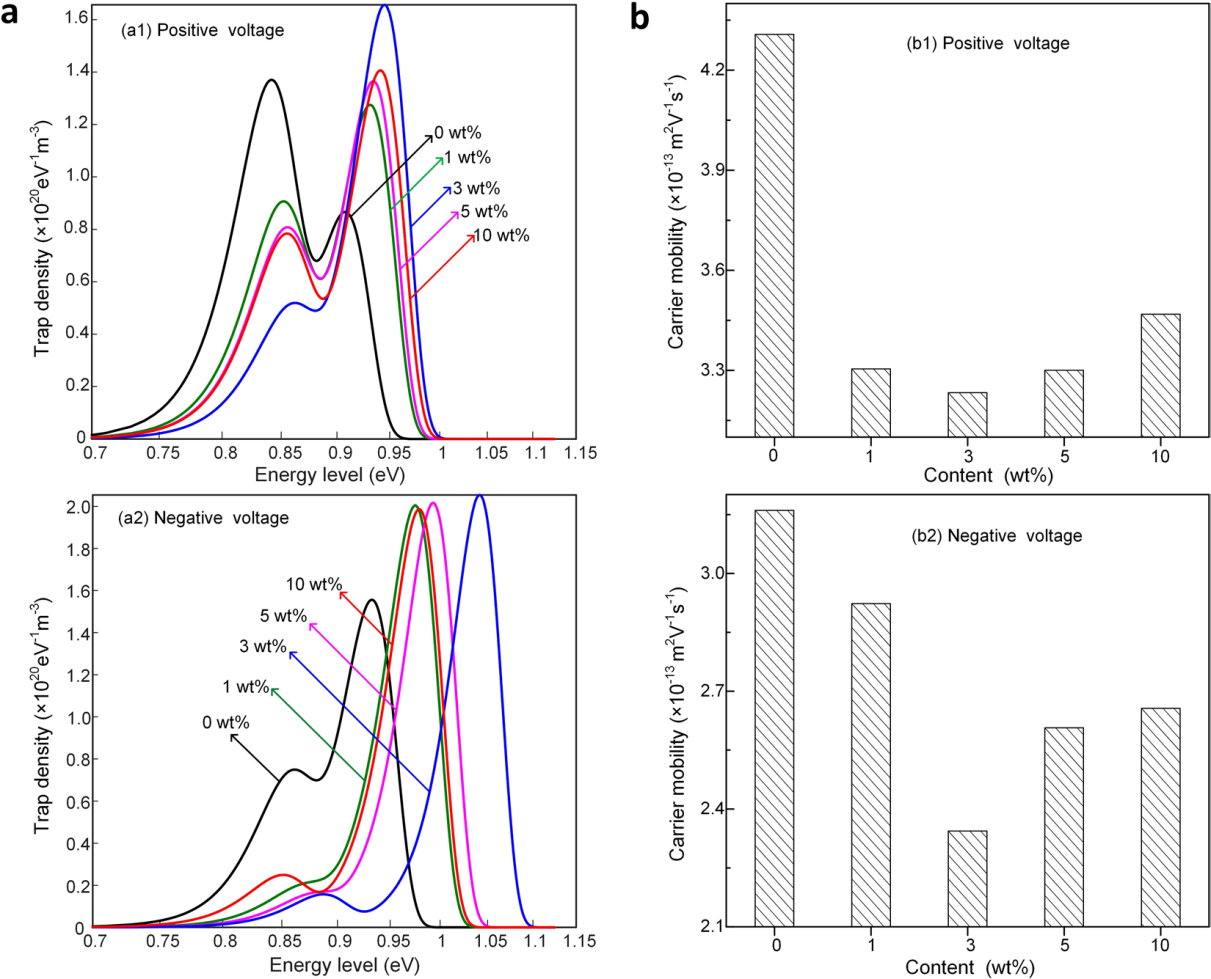
(**a**) Trap distribution behaviors of EPDM/OVPOSS measured by SPD method. (**b**) Carrier mobility behaviors of EPDM/OVPOSS measured by SPD method.

Fig. 6a shows the relationship between the trap density and the corresponding trap energy level of EPDM/OVPOSS measured by SPD method. In Fig. 6a1, two peaks are obtained in the computed results, which indicate that two types of decay processes are simultaneously occurred by controlling two different behaviors of charges. The peak located at shallow (or lower) trap energy level represents the maximum value of shallow trapped charge density and the peak located at deep (or higher) trap energy level represents the deep one. It indicates that the deep trapped charge density generally become larger when OVPOSS is filled into the EPDM. When the OVPOSS content is increased from 0 wt% to 3wt %, the deep trapped charges are located from 0.9 eV to 0.95 eV. However, when the OVPOSS content continues increasing to 5 wt%, the deep trapped charges tend to be located at smaller trap energy level. As it is shown in the SEM, the OVPOSS is aggregated in different patterns. The distance between two particles becomes larger in some regions, so the carriers can easily pass through this region without falling into the trap. Furthermore, the aggregation of the OVPOSS can lead to the overlap of electrostatic potential between the particles to weaken the deep energy level effect on the carrier mobility. When the OVPOSS content continues increasing to 10 wt%, there is a little increase of trap density and energy level. It is presumed that the negative electrostatic potential effects become larger as the aggregated particle size reaches ~1 μm. However, the aggregated particles can also introduce physical defects, which enhance the carrier mobility to facilitate the electrical tree growth.

The tendency of the carrier mobility with different OVPOSS content is shown in Fig. 6b1. The captured charges are more difficult to escape from deep trapped charge level than the shallow ones^38^. As the OVPOSS is filled into EPDM, the deep trapped charge level gets higher. Therefore, the charges captured by the deep trapped charge level are more difficult to escape and transport to the ground electrode, which result in the smaller carrier mobility in the filled samples. When the OVPOSS content is 3 wt %, the deepest trapped charge level and largest trapped charge density greatly restrain the destruction of the molecular chain by reducing the carrier mobility to the smallest during the electrical tree propagation process. As the electrical tree grows, some electrical tree channels can be carbonized, which transport charges to end section of carbonized channels in the form of conduct current and enhance the high electric field stress. When the electric field stress reaches the threshold value, partial discharges are generated to erode or extent the main channels. When OVPOSS is filled into the EPDM, the deep trapped charge level can capture the transported charges in the carbonized electrical tree channels to reduce the conduct current, which relieves the electric field stress at end section of carbonized channels.

The hole and electron carriers are shown different trapping and detrapping characteristics, which have been verified by both simulation and experiment methods^39^. The energy level distribution of electron-type traps in EPDM/OVPOSS is quite different from the hole-type ones. The deep trapped charge level of hole-type traps is located 0.9–0.95 eV and it is 0.93–1.04 eV for electron-type ones. The mean value of deep trapped charge level for electron-type traps is a little larger than the hole-type ones. Moreover, the deep trapped charge density of electron-type is higher than the hole type ones, whereas the shallow trapped charge density of electron-type is much lower compared with the hole-type ones. As the energy level becomes deeper and the trap density turns to be larger in the filled samples, the injected charges are captured by deeper energy level to form a steady state. Therefore, less injected charges are fallen into the shallower energy level and the apparent shallower trap density is decreased with the increase of POSS content. When the electrical tree is propagated under negative pulse voltage, the injected charges from the needle tip or hot electrons excited via an Auger-type process are captured by electron-type traps. The energy level and trap density of electron-type deep trapped charges are larger than the hole-type ones, resulting in a smaller carrier mobility with the negative voltage. Thus, the electrical tree are more difficult to propagate by collision with the molecular chain when the negative pulse voltage is stressed on the samples.

### 3D electrostatic potential and DOS behaviors

**Figure 7:**
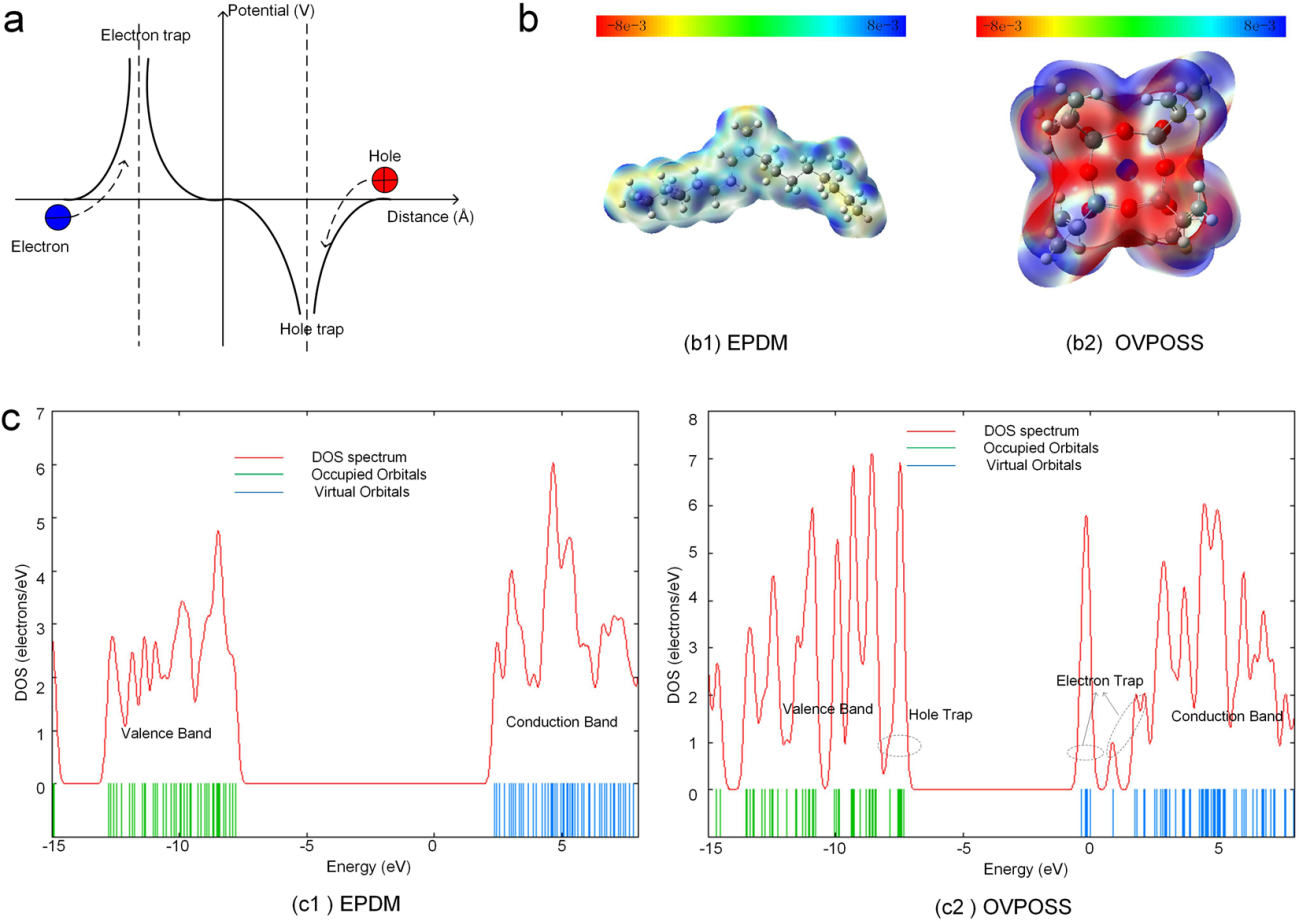
(**a**) Relationship between potential and charge trapping. (**b**) 3D electrostatic potential distribution in EPDM and OVPOSS. (**c**) DOS and energy level in EPDM and OVPOSS.

According the above experimental results, the transportation of charge carriers in 3 wt% EPDM/OVPOSS is significantly suppressed by introducing deeper trapped charge level, resulting in a lower electrical tree inception probability, a shorter tree length and a slower growth speed than the neat EPDM. It is believed that the trap distribution is closely associated with the electrostatic potential in polymer. The positive electrostatic potential captures electrons and transforms to electron-type traps, while the negative electrostatic potential captures holes and transforms to hole-type traps as shown in Fig. 7a. Fig. 7b shows the 3D electrostatic potential distribution in EPDM and OVPOSS. ENB (Ethylidene Norbornene) is not included in the EDPM model, considering the less content in unit molecular chain. In the 3D electrostatic potential distribution, the warm color shows negatively charged and the cold color shows positively charged, respectively. As the electronegativity between carbon of the main chain and hydrogen of the side chain is different, the orbital electron is biased in EPDM. The carbon of the main chain and the hydrogen of the side chain are respectively negatively and positively charged in EPDM, as shown in Fig. 7b1. Therefore, hot electrons are not easier close to the main chain since they are repelled to enter the outside of the hydrogen orbital in the side chain. It was reported that chemical groups such as conjugated carbon double bonds (C=C) could create a trap site by inherent (permanent) dipole moments which generated an electric potential well to capture charge carriers in polymer film^40,41^. In OVPOSS, the hydrogen in conjugated carbon double bonds (C=C) of the organic side-groups is positively charged and the oxygen of the main cage skeleton is negatively charged. It is interesting to find that the center of the main cage skeleton is positively charged, which generates electron-type traps to capture electrons.

Fig. 7c shows electronic energy level and DOS (Density of States) in EPDM and OVPOSS. In EPDM, the lowest unoccupied molecular orbital is 2.37 eV, the highest occupied molecular orbital is −7.8 eV, and the energy gap is 10.17 eV. In OVPOSS, the lowest unoccupied molecular orbital is −0.38 eV, the highest occupied molecular orbital is −7.34 eV, and the energy gap is 6.96 eV. According to DOS spectrum, OVPOSS generates new hole traps in sample as the highest occupied molecular orbital is higher than EPDM ones. Also, new electron traps are imported by OVPOSS as the lowest unoccupied molecular orbital is lower than EPDM ones. It is found that only one hole trap of OVPOSS is introduced into EPDM, while there are three electron ones. The different behaviors between hole and electron traps of OVPOSS introduced into EPDM can explain energy level differences between electron-type and hole-type deep trapped charge characteristics in EPDM/OVPOSS. As the higher energy level of electron-type deep trapped charges is introduced into the filled samples than the hole-type ones, the hot electrons or injected charges are easier to be captured during the electrical tree propagation process. Thus, electrical tree is more difficult to propagate as negative voltage is applied on the sample than the positive voltage ones.

## Conclusion

In this paper, the effects of OVPOSS on the morphologies, inception probability and propagation process of electrical tree are investigated in EPDM/OVPOSS composites, and the trap distribution and carrier mobility behaviors of the samples are analyzed. The EPDM/OVPOSS with a filler content of 3 wt% has a significantly lower tree inception probability and a shorter tree length than the neat EPDM, which presents that the propagation of electrical tree is suppressed by the well-distributed OVPOSS. The deep trapped charge level is higher and deep trapped charge density is larger in 3 wt% EPDM/OVPOSS composites than the neat EPDM ones, which can reduce the charge transportation during the electrical treeing process. According to the simulation of 3D electrostatic potential and DOS behaviors in OVPOSS, it is proposed that the special electrostatic potential of OVPOSS can capture charge carriers and introduce deeper trapped charge level into EPDM to suppress the transportation of charge carriers, thus restraining the degradation of molecular bonds. However, much worse aggregation of OVPOSS is caused by further increasing of the filler content, which leads to the overlap of electrostatic potential to weaken the deep trap effect on the carrier mobility, thus electrical tree propagation is accelerated. Compared with the energy level of hole-type deep trapped charges, the electron-type one is larger, which can effectively capture charge carriers to reduce the collision with the molecular chain. Thus, electrical tree is more difficult to propagate when negative pulse voltage is stressed on the samples than the positive voltage one. In conclusion, the deeper trapped charge level and larger trapped charge density introduced by OVPOSS can modulate charge carrier transport, which are benefit to restrain electrical tree propagation and improve the lifetime of polymer dielectrics.

## Methods

### Preparation of EPDM/OVPOSS

EPDM (Nordel 4725) with an ethylene content of 70 wt.%, a propylene content of 25 wt.% and an ethylidenenorbornene content of 5 wt.% is supplied by Dow Chemical Company (USA). The OVPOSS with a purity of ≥98% are purchased from Zhengzhou Alfachem Co., LTD (China). The 2,5-dimethyl-2,5-di-(tert-butylperoxy)-hexane is employed as the cross-linking agent. The OVPOSS with different filler content of 0 wt%, 1 wt%, 3 wt%, 5 wt% and 10 wt% are mechanically mixed with EPDM at the processing temperature of 458 K before the cross-linking agent is added. The mixed compounds are hot-pressed in a stainless steel mold at 443 K under a pressure of 15 MPa for 10 minutes. The obtained samples with size of 100 × 25 × 4 mm^3^ or 100 × 100 × 0.300 mm^3^ are post-cured at 473 K for 4 h under ambient pressure.

### Electrical tree degradation tests

**Figure 8:**
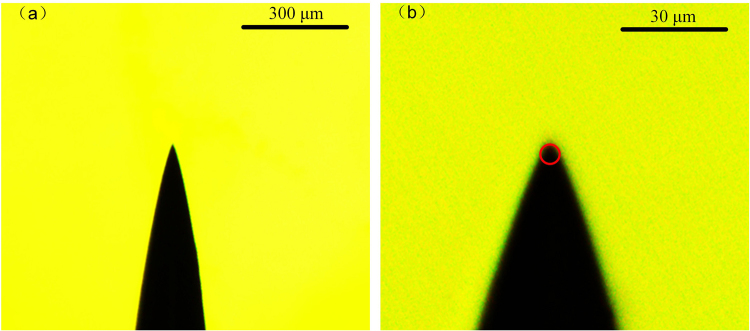
Microstructure of the needle electrode.

In the test, the sample is stressed on a needle-plane electrode system. The microstructure of the steel needle electrode is shown in Fig. 8, in which the curvature of the needle electrode is about 3 μm. The distance between the needle electrode and the ground electrode is 2 mm, which is controlled by the observation of the electron microscope. The experimental system consists of a power source system to trigger the electrical tree, an image processing system to capture the image during the electrical treeing process. Power source system is a high voltage generator, which can provide repetitive pulse voltage with pulse amplitude ranging from 0 to 30 kV. The pulse frequency is 200 Hz in the experiment. The image processing system is consisted of a digital monitoring system (SDK-2000), a computer and a controlled light source.

### SPD experiments

A typical needle-grid-ground is employed to charge thin films, which is composed of a needle electrode, a mesh grid and a ground electrode^42^. The surface charge characteristic test is carried out at 25 °C with relative humidity of ~30%. In this experiment, the needle electrode with the diameter of 1 mm and tip radius of ~15 μm is set 5 mm above the mesh grid. The distance between the mesh grid and the sample is 5 mm. The charging time is set as 5 minutes, and after charged, the sample is quickly moved to the Kelvin probe (TREK-6000B-5C), which is positioned 3 mm above the sample. An electrostatic voltmeter (TREK MODEL 347–3HCE) connected to the probe is employed to measure the surface potential.

The electrical treeing is closely related to the trapping and de-trapping behaviors of carriers, and both the carrier mobility and space charge accumulation of the polymer materials are determined by trap distribution characteristics^35^. Deep traps can reduce the kinetic energy of charge carriers by capturing them to result in a decrease of charge mobility. Both deep trapped charge level and density are benefit to enhance the breakdown strength^23^. The surface potential decay (SPD) technique is employed as a useful method to investigate the charge transportation and trap distribution behaviors^42^. The *tdV/dt* characteristics are associated with the time of the captured charge to escape from the traps. The relationship between the trap density (*N*_*t*_) and trap energy level (*E*_*t*_) can be expressed as^43^:

4 $${N}_{t}=\frac{{\varepsilon }_{0}\cdot {\varepsilon }_{r}}{{q}_{e}\cdot L}\cdot t\frac{dV}{dt}$$

5 $${E}_{t}=kTln(v\cdot t)$$

where *ε*_0_*, ε*_*r*_ are the vacuum dielectric constant and relative dielectric constant, *q*_*e*_ is the coulomb’s quantity of electron, *L* is the thickness of the sample, *V* is the surface potential, *t* is the decay time of the surface potential, *K* the Boltzmann’s constant, *T* is the temperature, υ is the attempt to escape frequency. In this paper, the distribution of trap energy level and the corresponding trap density are calculated and summarized to analyze the electrical property behaviors of EPDM/OVPOSS composite.

The carrier mobility of the polymer dielectric is depended on the trap depth and density, the higher trap energy level and trap density often result in the lower carrier mobility. During partial discharging process of electrical tree propagation, the hot electrons in lower trap energy level are more easily released and transported with a higher effective mobility to the ground electrodes, inducing a severer collision with molecular chains or a larger conduction current. The carrier mobility *μ* can be obtained by,

6 $$\mu =\frac{{L}^{2}}{{t}_{({\rm{T}})}\times {V}_{0}}$$

where *L* is the thickness of the sample, *V*_0_ is the initial surface potential and *t*_(T)_ is the transit time^38^. Through the calculation of carrier mobility, the characteristics of charge transportation can be obtained and comprehended.

### 3D electrostatic potential and DOS simulation

The 3D electrostatic potential distribution, electronic energy level and DOS (Density of States) of EPDM and OVPOSS molecular models are determined by density functional theory (DFT). DFT is based on the first-principle calculation and the solution of basic Schrodinger’s equation to determine the wave functions^44^. Molecular orbitals (MOs), which are constructed by combining the atomic orbitals and the hybrid orbitals from each atom of the molecular, are obtained by the self-consistent (SCF) method. In this paper, DFT methods with the B3LYP hybrid functional and the 6–31 G (d) basis function are selected to obtain 3D electrostatic potential distribution, electronic energy level and DOS^45^.
